# Using apple watch ECG data for heart rate variability monitoring and stress prediction: A pilot study

**DOI:** 10.3389/fdgth.2022.1058826

**Published:** 2022-12-09

**Authors:** Pedro Elkind Velmovitsky, Paulo Alencar, Scott T. Leatherdale, Donald Cowan, Plinio Pelegrini Morita

**Affiliations:** ^1^ School of Public Health and Health Systems, University of Waterloo, Waterloo, ON, Canada; ^2^ David R. Cheriton School of Computer Science, University of Waterloo, Waterloo, ON, Canada; ^3^ Research Institute for Aging, University of Waterloo, Waterloo, ON, Canada; ^4^ Department of Systems Design Engineering, University of Waterloo, Waterloo, ON, Canada; ^5^ Institute of Health Policy, Management, and Evaluation, Dalla Lana School of Public Health, University of Toronto, Toronto, ON, Canada; ^6^ Centre for Digital Therapeutics, Techna Institute, University Health Network, Toronto, ON, Canada

**Keywords:** mHealth, stress, machine learning, mobile, wearable, ECG, Apple watch

## Abstract

Stress is an increasingly prevalent mental health condition that can have serious effects on human health. The development of stress prediction tools would greatly benefit public health by allowing policy initiatives and early stress-reducing interventions. The advent of mobile health technologies including smartphones and smartwatches has made it possible to collect objective, real-time, and continuous health data. We sought to pilot the collection of heart rate variability data from the Apple Watch electrocardiograph (ECG) sensor and apply machine learning techniques to develop a stress prediction tool. Random Forest (RF) and Support Vector Machines (SVM) were used to model stress based on ECG measurements and stress questionnaire data collected from 33 study participants. Data were stratified into socio-demographic classes to further explore our prediction model. Overall, the RF model performed slightly better than SVM, with results having an accuracy within the low end of state-of-the-art. Our models showed specificity in their capacity to assess “no stress” states but were less successful at capturing “stress” states. Overall, the results presented here suggest that, with further development and refinement, Apple Watch ECG sensor data could be used to develop a stress prediction tool. A wearable device capable of continuous, real-time stress monitoring would enable individuals to respond early to changes in their mental health. Furthermore, large-scale data collection from such devices would inform public health initiatives and policies.

## Introduction

Stress is an often overlooked determinant of health. High stress levels are linked to severe health problems such as depression, obesity, and cardiovascular diseases (1). Unfortunately, 1 in 5 Canadian citizens report experiencing high levels of stress daily (2). Increased awareness of mental health has emphasized the need for more timely stress monitoring and early intervention, and the collection of population-wide stress data could support public health initiatives and interventions.

Self-reporting continues to be the gold standard for monitoring stress. These methods face challenges and limitations such as social and recall bias (3, 4), loss due to follow-up (3, 4), delays between collection and reporting (5), and costs/logistics (3, 5). However, the link between stress and multiple biomarkers has revealed opportunities to develop technologies to quantify stress. One such feature is heart rate variability (HRV) which is now routinely quantified through an electrocardiograph (ECG). ECGs have been widely used for stress prediction, and are typically performed at a healthcare facility which limits their accessibility. The development of rapid point-of-care or self-monitoring devices would improve patient outcomes, providing invaluable information for public health agencies and real-time interventions (e.g., guided meditations) (6, 7).

Digital technologies, including smartphones and wearable smartwatches, are pervasive in our lives. In 2020, the number of Apple Watch users worldwide was estimated at 100 million (8). In line with the modern health trend toward patient self-care, these technologies now include sensors designed to continuously collect health data with minimal user effort (9). Collected health parameters include steps, heart rate, blood pressure, and sleep. These technologies now generate massive quantities of objective data. Further, the datasets obtained from this novel, real-life data can be used to create prediction models using Machine Learning (ML), allowing public health agencies to better understand and study the prevalence of a condition in a population.

Apple Health, a popular source of digital health data, has recently introduced an ECG sensor to their Apple Watch device (7, 10). The sensor, which is similar to a 1-lead ECG, collects 30 s of data through an electrode placed on the device's digital crown (11). According to Apple, studies have shown good agreement in classifying the rhythm of the Apple Watch ECG compared to standard 12-lead ECGs, and in a clinical trial of 600 participants the ECG sensor had 99.6% specificity when classifying synus rhythm and 98.3% sensitivity for atrial fibrillation (10).

ECG data collected from this wearable device could potentially be employed to predict stress: users would simply take a non-invasive 30-second ECG and get instant feedback on their stress levels. It is currently unclear whether the brief 30-second ECG reading will be sufficient for stress prediction.

The goal of this work was to pilot the use of Apple Watch ECG data for stress prediction. This analysis is part of the development of a Mobile Health Platform (MHP), which collects Apple Health data from several mobile and wearable devices (6, 7). We collected ECG and stress questionnaire data from 36 participants over 2 weeks with the platform. We applied the machine learning models Random Forests (RFs) and Support Vector Machines (SVMs), as these models have been successfully used in stress prediction literature (12). To the best of our knowledge, this is the first work that utilizes Apple Watch ECG for stress prediction. We found that the models performed at the low end of the state-of-the-art stress prediction technology. We were able to identify several HRV features, as well as socio-demographic classes which impacted the accuracy of the model. The results suggest that, with further development, Apple Watch ECG sensors could be employed for mobile, real-time stress prediction.

### Related work

The authors of Can et al. (12) provide a survey of stress prediction in real-life scenarios with mobile health technologies. As can be seen in this survey, and supported by stress prediction literature, successful methods for stress detection are Random Forests (RFs) and Support Vector Machines (SVMs), which were selected for this study.

Examples of studies that use these methods include Hovsepian et al. (13), which trains an SVM using ECG and respiration data in both laboratory and real-life settings. The model outputs the probability that a user is stressed with an accuracy of 90% in the laboratory and 72% in real-life. Muaremi et al. (14) collected ECG, respiration, galvanic skin response, sleep data and posture of sleeping individuals, achieving good accuracy with SVMs (73%) and RF (71%). Gjoreski et al. (15) use laboratory data to build RFs that predict stress with an accuracy of 83%; then, the RF model is used as an output to train an SVM that achieves 76% accuracy on real-life data. Can et al. (16) used heart rate variability and electrodermal activity data for real-life stress prediction, achieving 68% accuracy with SVM and 66% with RF. Based on these considerations and review results (12), the state-of-the-art accuracy for stress detection in real-life settings lies approximately between 60% and 80%.

Regarding HRV analyses, the Task Force of The European Society of Cardiology and the North American Society of Pacing and Electrophysiology provides guidelines on the measurement and analyses of HRV data (17), which were of great help for this work (more details are described in the sections below). Further, Acharya et al. provide a review of HRV metrics and their meaning (18), while Benchekroun et al. analyze the impact of missing data on several metrics and studied different interpolation techniques to handle missing data (19). Baek et al. analyzed several of these metrics on ultra-short term measurements and defines the minimum time interval for each of these metrics to be valid when compared to standard measurements, and found that each metric is different with some requiring only a few seconds of data while others require several minutes (20). The same work also showed that HRV can vary according to factors such as age (20).

## Materials and methods

### Data collection

**Figure 1:**
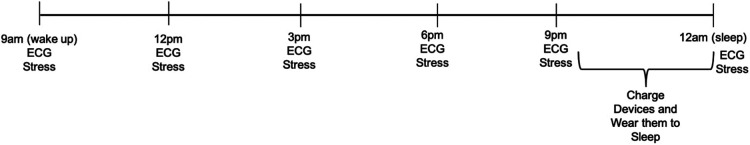
Study protocol.

Participants were given an iPhone 7 with iOS 15.0 and an Apple Watch Series 6 containing an installed Apple Watch ECG app (WatchOS 8.3) for two weeks. Following the Ecological Momentary Assessment (EMA) methodology (13), which enables self-reporting to approximate real-life scenarios, users were asked to perform an ECG reading using the app. EMAs are further described in section 2.3. They were instructed to collect data 6 times during the day in approximately three-hour intervals. Before the ECG collection, participants were asked to complete a stress questionnaire on the iPhone using the MHP. Figure 1 shows the study protocol (the times are just reference; participants were asked to start measurements at wake-up).

### Apple watch ECG application

WatchOS 8.3 is an application capable of recording ECG measurements *via* an Apple Watch version 4 or higher. Briefly, ECG measurements requires users to open the ECG app and place their finger on the digital crown of the device and remain still for 30 s (14). The instructions distributed to the users can be found in the Supplementary Material. ECG readings were automatically stored in Apple's HealthKit API. We extracted the API data through the MHP and saved it in JSON format on our database.

### Stress questionnaires

There are a limited number of validated stress questionnaires for EMA-style data collection. To mitigate this issue, we used the stress subscale of the Depression, Anxiety, and Stress Scale (DASS-21) as there is promising evidence of using DASS-21 with EMA (Questions 1–7) (15). This was combined with a single-item measure (Question 8) used successfully for stress measurement with a moderate correlation to robust stress questionnaires (16).

The following 8 questions, on a LIKERT-type scale (15), were designated as the Stress Questionnaire for participants:

1. I felt that I was using a lot of nervous energy;
2. I found it hard to wind down;
3. I found myself getting agitated;
4. I found it difficult to relax;
5. I tended to over-react to situations;
6. I was intolerant of anything that kept me from getting on with what I was doing;
7. I felt that I was rather touchy;
8. Right now, I am …

Questions 1–7 have the options: “Not at all”, “To some degree”, “To a considerable degree”, and “Very much”, while Question 8 has “Stressed Out”, “Definitely stressed”, “A little stressed”, “Feeling good”, and “Feeling great”. The questions were displayed to the user in a random order each time the questionnaire is filled.

### Mobile health platform

As discussed, we developed a mobile health platform (MHP) using Apple's software for creating iOS apps, (XCode, version 12.5.1). The MHP acted as a user interface: automatically collecting data from Apple Health (*via* HealthKit) and allowing users to complete the stress questionnaire (17). More details are provided in the Results section.

### Study population

Participants were recruited from the University of Waterloo (students) and online advertisements (workers; Facebook Ads and Kijiji). Participants were local to the Kitchener-Waterloo region in Ontario, Canada. Participants were initially only included if they were healthy. This requirement was subsequently relaxed to allow “unhealthy” participants (chronic disease or illness, prescription drug use, or frequent use of alcohol or drugs). Participants were offered CAD 100.00 for two weeks of data collection. Additional data collection beyond two weeks was requested from some participants who had missed measurements (less than 6 measurements per day). This study was approved by University Waterloo Research Ethics Board [REB (43612)]. Participant consent for data collection was obtained before device distribution. Data from 40 participants were collected. After applying the data cleaning and pre-processing described below, 7 participants had less than 50% of data points available. Therefore, these participants were excluded, and the subsequent analysis was done on 33 participants. Table 1 shows the characteristics of the study participants. Of note, 27% of participants were male and 73% were female; 24% were aged 18–24, 30% were aged 25–34 and the same proportion was found for participants aged 35–44, 12% were aged 45–64, and only 3% (1 participant) was aged over 65. The average BMI was 27.2 (±6.70), and participants had an average of 65.1 (±11.80) valid ECG recordings.

**Table 1:** Study population characteristics.

Participants (*N* = 33)	Frequency	Percentage
Age		
18–24	8	24
25–34	10	30
35–44	10	30
45–64	4	12
Above 65	1	3
Gender		
Male	9	27
Female	24	73
SES		
Low (0–$30,000)	15	45
Medium ($30,000–$100,000)	15	45
High (Above $100,000)	2	6
Do not wish to disclose	1	3
Profession		
Full-time	14	42
Part-time	3	9
Student	13	39
Self-employed/Other	2	6
Retired	1	3
Ethnicity		
Black or African American	2	6
Chinese	4	12
Indian	1	3
Latin American	8	24
South Asian	6	18
White	12	36
Health Status		
Healthy	26	79
Chronic Disease or Illness, Prescription Drug Use, Smoking or Alcohol	7	21

### Data pre-processing and analysis

We exported the ECG data from HealthKit into a CSV format and sorted each ECG voltage measurement by timestamps. We removed any ECG measurement that was classified as Poor Recording or Inconclusive by the ECG app (10). The CSV file was imported into Kubios Premium 3.5.0 to determine heart rate variability (HRV) signals (18, 19).

In order to apply signal filtering, we used the Kubios automatic beat detection feature as well as automatic noise detection, which excludes all segments marked as noise—the default Medium setting was used for noise segments. Kubios also has an automatic artefact correction method which was used for this analysis, and any samples containing more than 5% of corrected beats was removed. A list of the features generated by Kubios is presented in Table 2 (18, 20). Kubios automatically calculates a list of features for HRV analysis (18, 20). However, some features could not be calculated by the software with the 30 s measurements, and so these features were not used. The full list of Kubios features used for the analyses are mentioned in Table 2.

**Table 2:** HRV features.

Name	Description
Time-Domain Features	
PNS Index	Parasympathetic nervous system activity compared to normal resting values
SNS Index	Sympathetic nervous system activity compared to normal resting values
Stress Index	Square root of Baevsky's stress index
Mean RR	Mean of R-R intervals
SDNN	Standard deviation of R-R intervals
Mean HR	Mean of heart rate
STD HR	Standard deviation of instantaneous heart rate
Min HR	Minimum instantaneous heart rate calculated using 5 beat moving average
Max HR	Maximum instantaneous heart rate calculated using 5 beat moving average
RMSSD	Square root of the mean squared differences between successive RR intervals
DC	Heart rate deceleration capacity
DCMod	Modified DC computer as a two-point difference
AC	Heart rate acceleration capacity
ACMod	Modified AC computer as a two-point difference
Frequency-Domain Features	
FFT LF	Fast Fourier Transform Low Frequency band components
FFT HF	Fast Fourier Transform High Frequency band components
AR LF	Autoregressive Low Frequency band components
AR HF	Autoregressive High Frequency band components
FFT Absolute Power LF	Fast Fourier Transform Absolute Power of Low Frequency band components
FFT Absolute Power HF	Fast Fourier Transform Absolute Power of High Frequency band components
AR Absolute Power LF	Autoregressive Absolute Power of Low Frequency band components
AR Absolute Power HF	Autoregressive Absolute Power of High Frequency band components
FFT Relative Power LF	Fast Fourier Transform Relative Power of Low Frequency band components
FFT Relative Power HF	Fast Fourier Transform Relative Power of High Frequency band components
AR Relative Power LF	Autoregressive Relative Power of Low Frequency band components
AR Relative Power HF	Autoregressive Relative Power of High Frequency band components
FFT Normalized Power LF	Fast Fourier Transform Normalized Power of Low Frequency band components
FFT Normalized Power HF	Fast Fourier Transform Normalized Power of High Frequency band components
FFT Total Power	Fast Fourier Transform Total Power
FFT LF/HF	Fast Fourier Transform ratio between low and high frequency
AR Normalized Power LF	Autoregressive Normalized Power of Low Frequency band components
AR Normalized Power HF	Autoregressive Normalized Power of High Frequency band components
AR Total Power	Autoregressive Total Power
AR LF/HF	Autoregressive ratio between low and high frequency
Non-Linear Features	
SD1	The standard deviation perpendicular to the line-of-identity in Poincaré plot
SD2	The standard deviation along the line-of-identity in Poincaré plot
SD2/SD1	Ratio between SD2 and SD1

In addition, several features were excluded following recommendations made by the Task Force of The European Society of Cardiology and the North American Society of Pacing and Electrophysiology (21): we removed pNN50 and NN50 as they are highly correlated with the RMSSD, and the RMSSD was preferred. The TINN, HRV Tri Index, VLF, and log measurements were removed as they were indicated for longer time periods than that measured here. Finally, features that were highly correlated were identified using the Pearson correlation method (*r* = 0.95) and removed.

Participant stress states for each measurement were determined based on the results of the stress questionnaires. Measurements were categorized as “stress” or “no stress” based on the following criteria. The scores of the DASS-21 questions (Questions 1–7) were summed together and multiplied by 2; if the score was greater than 14, the sample was classified as “stress” (22). For the single-item measure (Question 8), the sample was classified as “stress” if the score was greater than 2. To integrate data from two separate questionnaires, if either the DASS-21 score or the single-item score was classified as “stress”, the sample was classified as “stress”.

We divided the dataset into 70% for training and validation and 30% for testing. We used 10-fold cross-validation for training the RFs and SVMs, which were developed using sci-kit learn. These models were chosen as they are widely and successfully used in stress prediction literature (12). The “GridSearchCV” function was used to tune the model parameters and find the best ones. The data were normalized using sci-kit learn's “StandardScaler” function for optimization.

The models were trained to the entire dataset as well as the subset of healthy participants. Given the relationship between HRV measures and demographics (23–26), we trained models based on age (18–24 years, 25–34 years, 35–44 years, and 45–65 years), gender (male, female), income (<$30,000 CAD, >$30,000 CAD), and profession (student, worker). For each model, we calculated feature importance for the RF model using the mean decrease in impurity (a 100% purity in a node means the decision tree's node contains only one class, and by assessing the difference between the impurity in the parent and child nodes we can calculate the best split in the tree and use it as a proxy for feature importance). For categories that had only one participant we did not perform the model analyses.

## Results

We sought to pilot the use of machine learning with Apple Watch ECG data as a step towards developing a wearable device for stress prediction. We recruited students and staff from the University of Waterloo (Ontario, Canada) to participate in a two-week study. Participants were given an iPhone 7 and an Apple Watch Series 6. It is important to note that this study is part of a larger wearable study involving other devices such as wireless blood pressure cuffs; for this study, we focus specifically on the iPhone and Apple Watch and on ECG measurements alone. The details of the other study are described elsewhere (6, 7).

**Figure 2:**
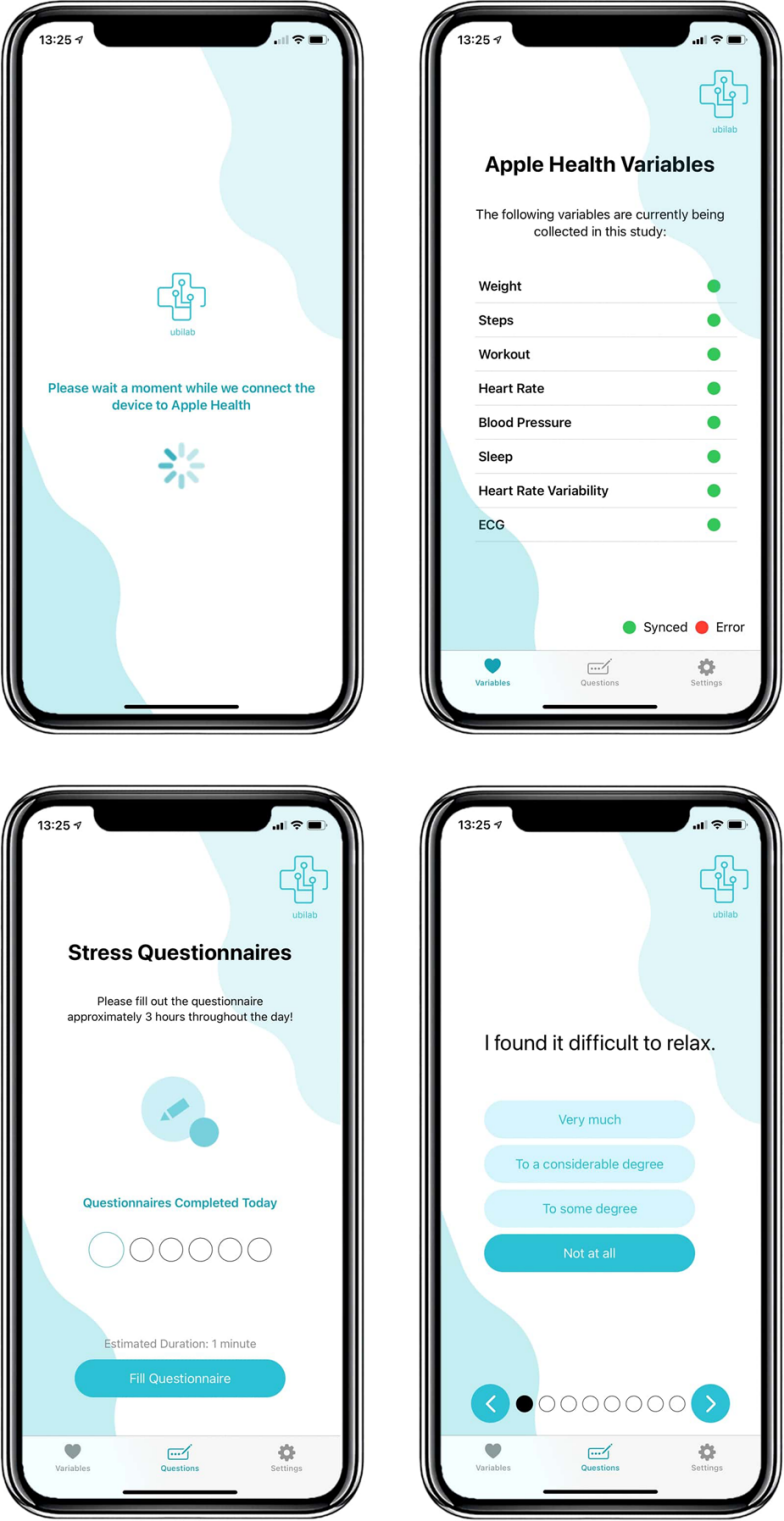
MHP interface.

Users were asked to collect ECG measures using the Apple Watch ECG app six times during the day at approximately three-hour intervals. Before the acquisition of each ECG, participants were asked to complete a stress questionnaire on the MHP developed for the study, which also updated new ECG measurements to our database. The MHP app interface is depicted in Figure 2.

As there are few validated stress questionnaires, we made use of the stress portion of the Depression, Anxiety, and Stress Scale (DASS-21) in conjunction with a single-item measure that has been used successfully in previous stress prediction studies (16). In total, we acquired 2421 ECG/survey measures from 33 participants after data cleaning and pre-processing. Readings were classified as “stress” or “no stress” based on the answers to the questionnaire (22). We applied the machine learning models Random Forests (RFs) and Support Vector Machines (SVMs) to train the model.

Table 3 shows a summary of the results for each trained model, described in more detail below.

**Table 3:** Metrics for each trained model.

	Random forest				Support vector machine			
	Items	Precision	Recall	F1-Score	Precision	Recall	F1-Score	Support
* *	*No Stress*	0.58	0.66	0.61	0.58	0.58	0.58	359
Complete Dataset	*Stress*	0.52	0.43	0.47	0.50	0.51	0.51	306
* *	*Accuracy*	–	–	0.55	–	–	0.54	665
* *	*Weighted Average*	0.55	0.55	0.55	0.54	0.54	0.54	665
* *	*No Stress*	0.59	0.73	0.65	0.57	0.57	0.57	283
Healthy Subjects	*Stress*	0.50	0.34	0.40	0.45	0.45	0.45	221
* *	*Accuracy*	–	–	0.56	–	–	0.52	504
* *	*Weighted Average*	0.55	0.56	0.54	0.52	0.52	0.52	504
* *	*No Stress*	0.64	0.88	0.74	0.63	0.75	0.69	95
Subjects Aged 18–24	*Stress*	0.50	0.19	0.27	0.43	0.31	0.36	59
	*Accuracy*	–	–	0.62	–	–	0.58	154
* *	*Weighted Average*	0.58	0.62	0.56	0.56	0.58	0.56	154
* *	*No Stress*	0.39	0.16	0.23	0.42	0.14	0.22	69
Subjects Aged 25–34	*Stress*	0.67	0.87	0.76	0.67	0.89	0.77	133
* *	*Accuracy*	–	–	0.63	–	–	0.64	202
* *	*Weighted Average*	0.57	0.63	0.58	0.58	0.64	0.58	202
* *	*No Stress*	0.69	0.91	0.78	0.68	0.67	0.68	116
Subjects Aged 35–44	*Stress*	0.61	0.26	0.37	0.43	0.45	0.44	65
* *	*Accuracy*	-	-	0.67	-	-	0.59	181
* *	*Weighted Average*	0.66	0.67	0.63	0.59	0.59	0.53	181
* *	*No Stress*	0.70	0.84	0.76	0.68	0.86	0.76	50
Subjects Aged 45–64	*Stress*	0.53	0.33	0.41	0.50	0.26	0.34	27
* *	*Accuracy*	–	–	0.66	–	–	0.65	77
* *	*Weighted Average*	0.64	0.66	0.64	0.62	0.65	0.61	77
* *	*No Stress*	0.64	0.68	0.66	0.57	0.56	0.57	95
Male Participants	*Stress*	0.62	0.58	0.60	0.52	0.53	0.52	85
* *	*Accuracy*	–	–	0.63	–	–	0.55	180
* *	*Weighted Average*	0.63	0.63	0.63	0.55	0.55	0.55	180
* *	*No Stress*	0.59	0.55	0.57	0.59	0.67	0.63	250
Female Participants	*Stress*	0.52	0.56	0.53	0.55	0.47	0.50	214
* *	*Accuracy*	–	–	0.55	–	–	0.58	464
* *	*Weighted Average*	0.56	0.55	0.55	0.57	0.58	0.57	464
* *	*No Stress*	0.62	0.74	0.68	0.64	0.63	0.64	180
Low SES Participants	*Stress*	0.45	0.32	0.37	0.45	0.45	0.45	118
* *	*Accuracy*	–	–	0.57	–	–	0.56	298
* *	*Weighted Average*	0.55	0.57	0.56	0.56	0.56	0.56	298
* *	*No Stress*	0.53	0.42	0.47	0.55	0.48	0.51	161
Medium and High SES Participants	*Stress*	0.53	0.64	0.58	0.55	0.62	0.58	167
* *	*Accuracy*	–	–	0.53	–	–	0.55	328
* *	*Weighted Average*	0.53	0.53	0.52	0.55	0.55	0.55	328
* *	*No Stress*	0.57	0.51	054	0.60	0.54	0.56	134
Students	*Stress*	0.54	0.60	0.57	0.56	0.62	0.59	128
* *	*Accuracy*	–	–	0.55	–	–	0.58	262
* *	*Weighted Average*	0.56	0.55	0.55	0.58	0.58	0.58	262
* *	*No Stress*	0.55	0.69	0.61	0.56	0.69	0.62	200
Workers	*Stress*	0.49	0.35	0.41	0.52	0.39	0.45	170
* *	*Accuracy*	–	–	0.53	–	–	0.55	370
* *	*Weighted Average*	0.52	0.53	0.52	0.54	0.55	0.54	370

### Stress prediction models using total dataset and subset with healthy subjects

The RF and SVM models were trained against the complete data set. The complete dataset was fairly balanced, with the “stress” class representing 46% of all test examples (306 out of 665 in the test dataset). Due to class imbalances, we reported the F1-score weighted. The best accuracy was achieved by the RF model with 55% compared to 54% for the SVM model (Table 3). Weighted averages were similar to the accuracy. Recall and precision were higher for the “no stress” class when compared to the “stress” class, with the SVM having a higher recall for the “stress” class than the RF. Results indicated that, when using ECG measurements from a wearable device in a real-life setting, both the RF and SVM machine learning models approached the lower end of state-of-the-art accuracy levels for predicting stress levels.

**Figure 3:**
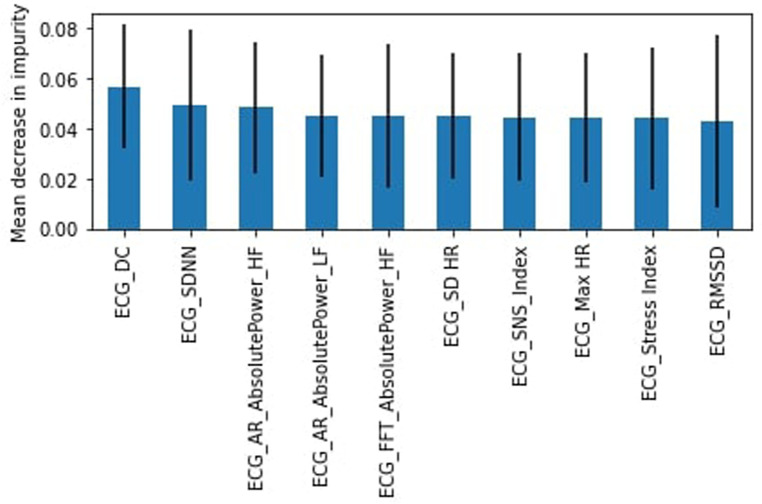
RF feature importance, complete dataset.

As there are multiple heart rate variability (HRV) parameters determined by the ECG test, we sought to identify the most important features for the RF algorithm. The top 10 features were determined using the mean decrease in impurity. Figure 3 shows that the top feature was the ECG heart rate deceleration capacity (DC) (Table 2).

**Figure 4:**
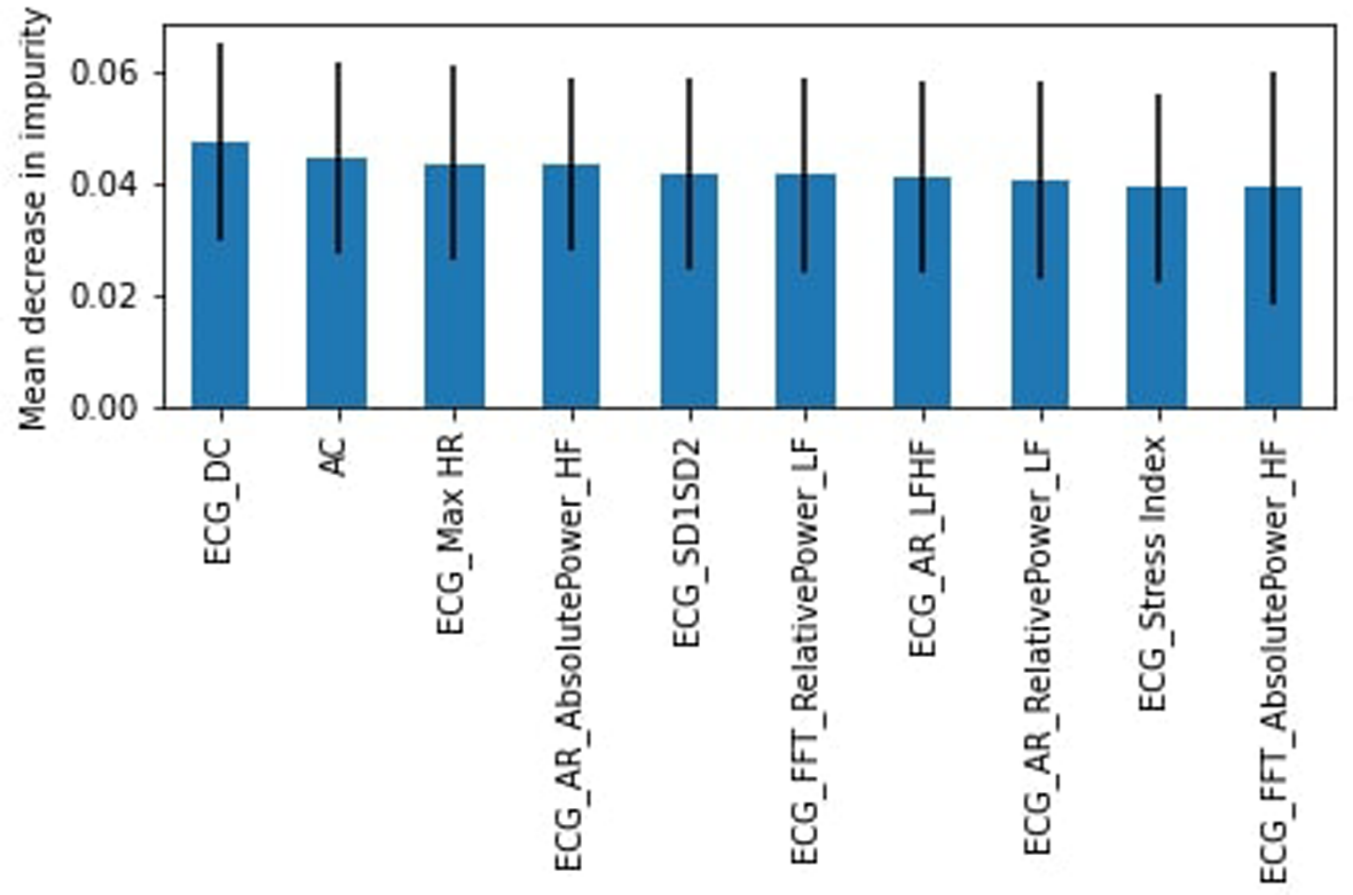
RF feature importance, healthy subjects.

Originally, all participants involved in this study were healthy; however, due to difficulty in finding study subjects, we relaxed the criteria to allow participants that were not healthy (chronic disease or illness, prescription drug use, or frequent use of alcohol or drugs). Again we found the RF model outperformed the SVM model. The healthy subset achieved a slightly lower weighted average for the “stress” class of 54% for the RF model with a recall of 34% (45% for SVM) and precision of 50%. DC was again identified as the most important feature (Figure 4) followed by the heart's acceleration capacity (AC).

### Impact of age on stress prediction models

Given that age influences HRV (21, 25), we trained the RF and SVM models based on age to see if we could improve the weighted average.

**Figure 5:**
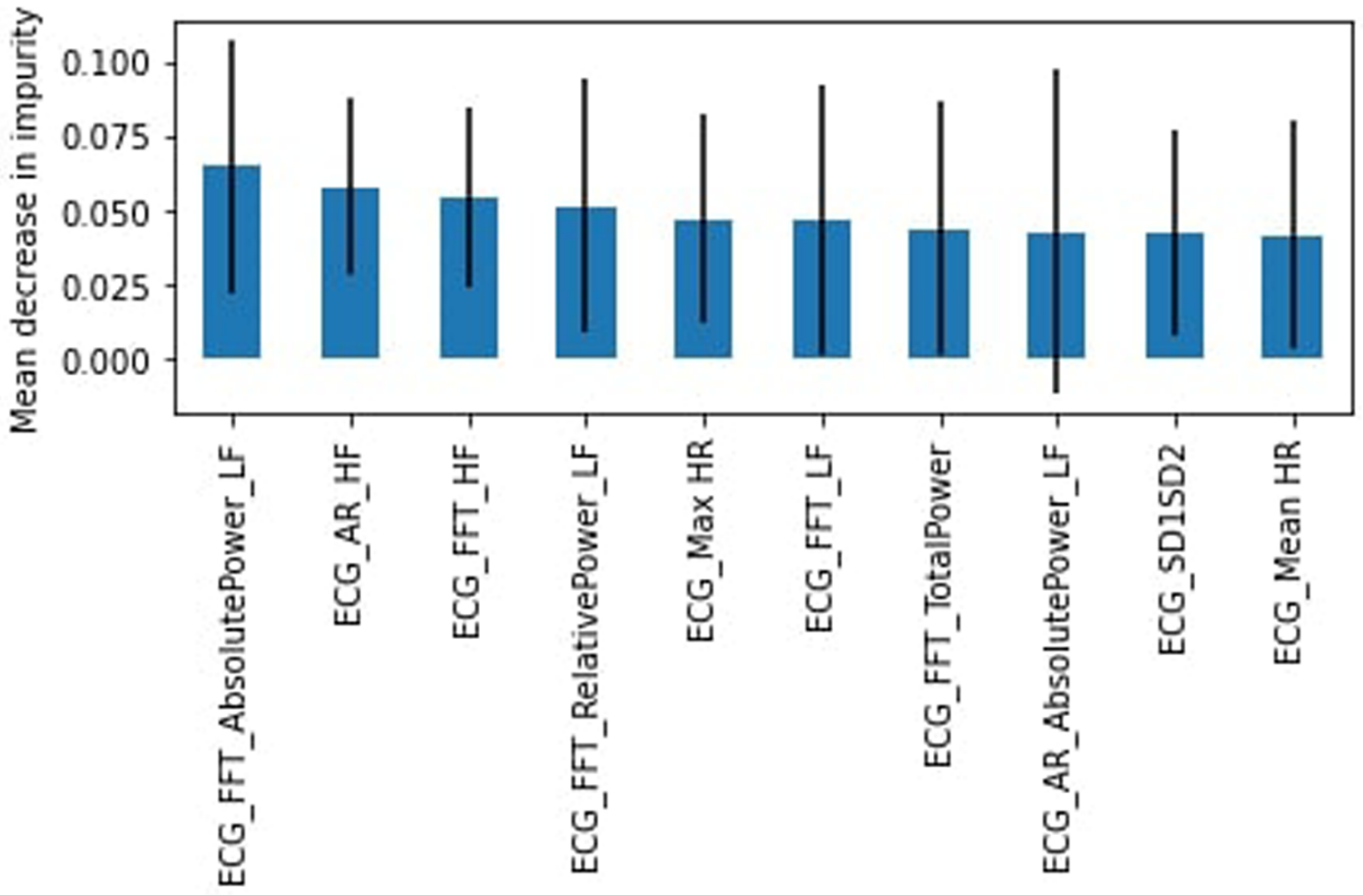
RF feature importance, subjects aged 18–24.

For the 18–24 years group, the “stress” class represented 38% of the data. The RF model outperformed the SVM model with an accuracy of 62% and an F1-score weighted of 56%. The recall and precision in the “stress” class were 19% and 50% respectively. Low Frequency Absolute Power calculated with FFT was identified as the most important feature (Figure 5).

**Figure 6:**
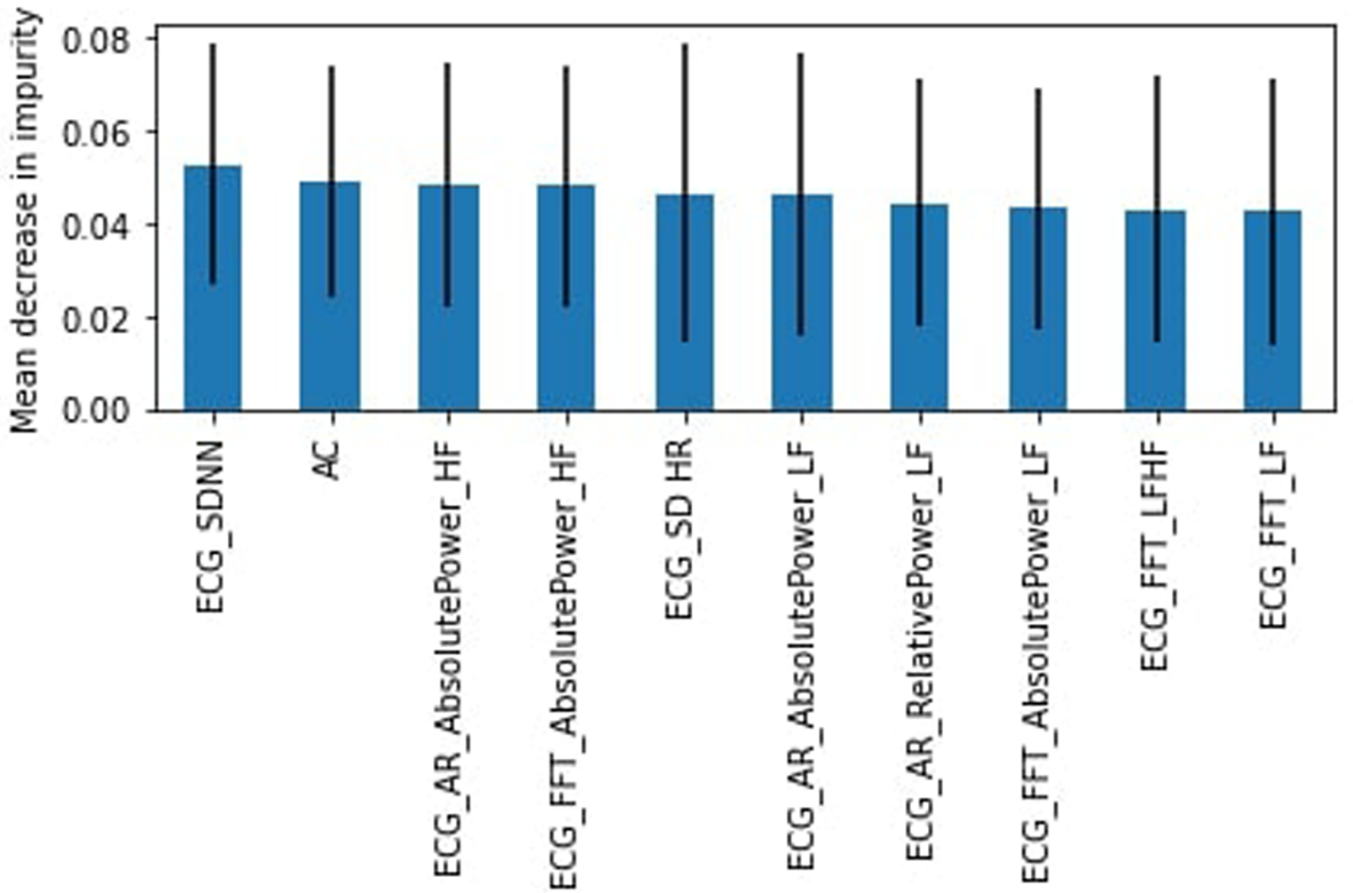
RF feature importance, subjects aged 25–34.

In the 25–34 years group, the “stress” class is the majority, representing 66% of the dataset. Here the SVM model slightly outperformed the RF model with an accuracy of 64% compared to 63%. The F1-score weighted was 58% for both models. In the “stress” category, the recall (87%) and precision (67%) were high but with a corresponding loss of recall (16%) and precision (39%) in the “no stress” class. The most important feature was the standard deviation of intervals, SDNN (Figure 6).

**Figure 7:**
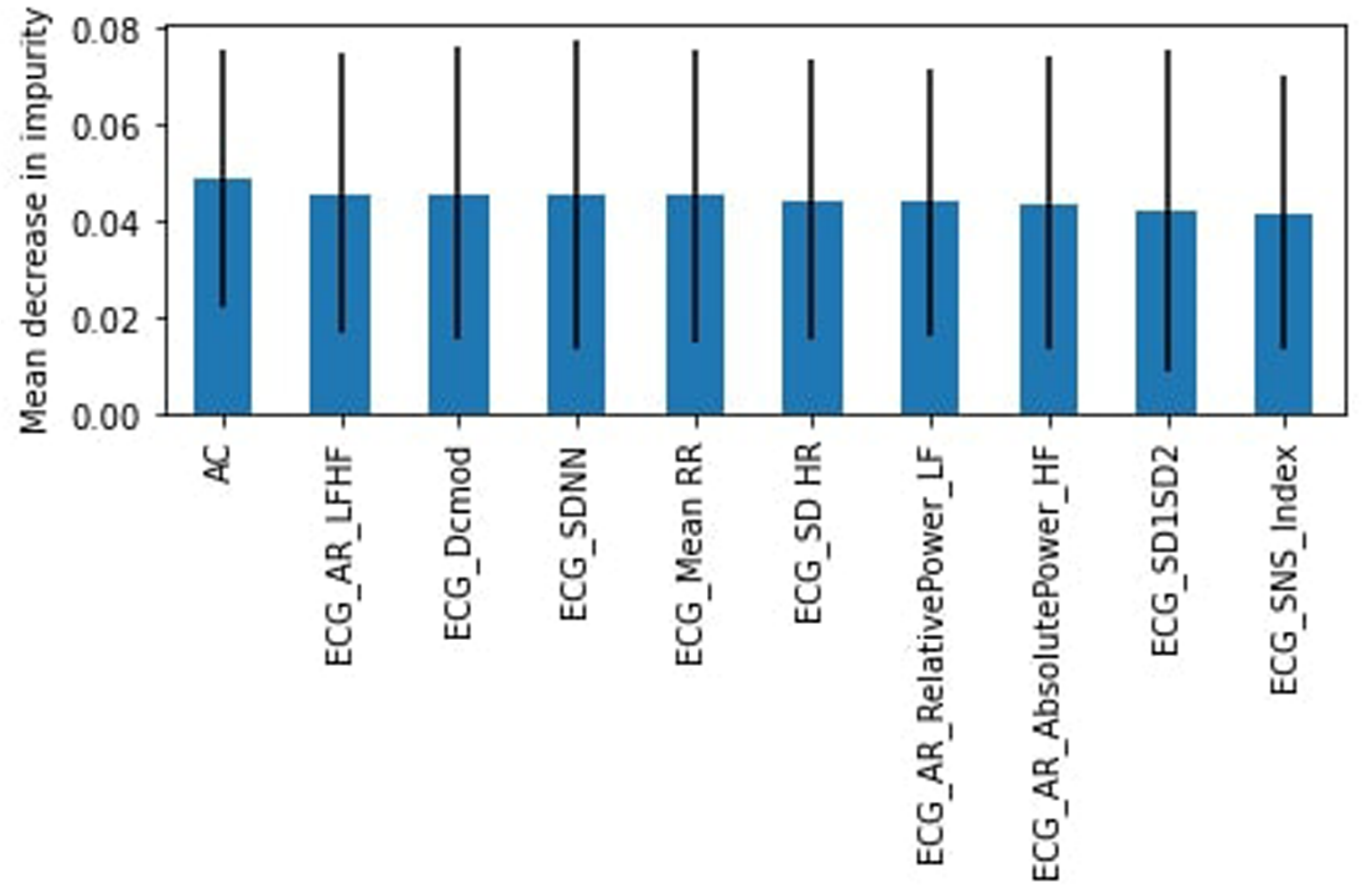
RF feature importance, subjects aged 35–44.

In the 35–44 years group, the “stress” class was the minority, representing 36% of the dataset. The RF had a higher accuracy of 67% (F1-score weighted of 63%) compared to 59% for the SVM model (F1-score weighted of 53). The SVM had higher recall than the RF for the “stress” class (45%–26%), but lower precision (43% to 61%). The AC was the most important feature (Figure 7).

**Figure 8:**
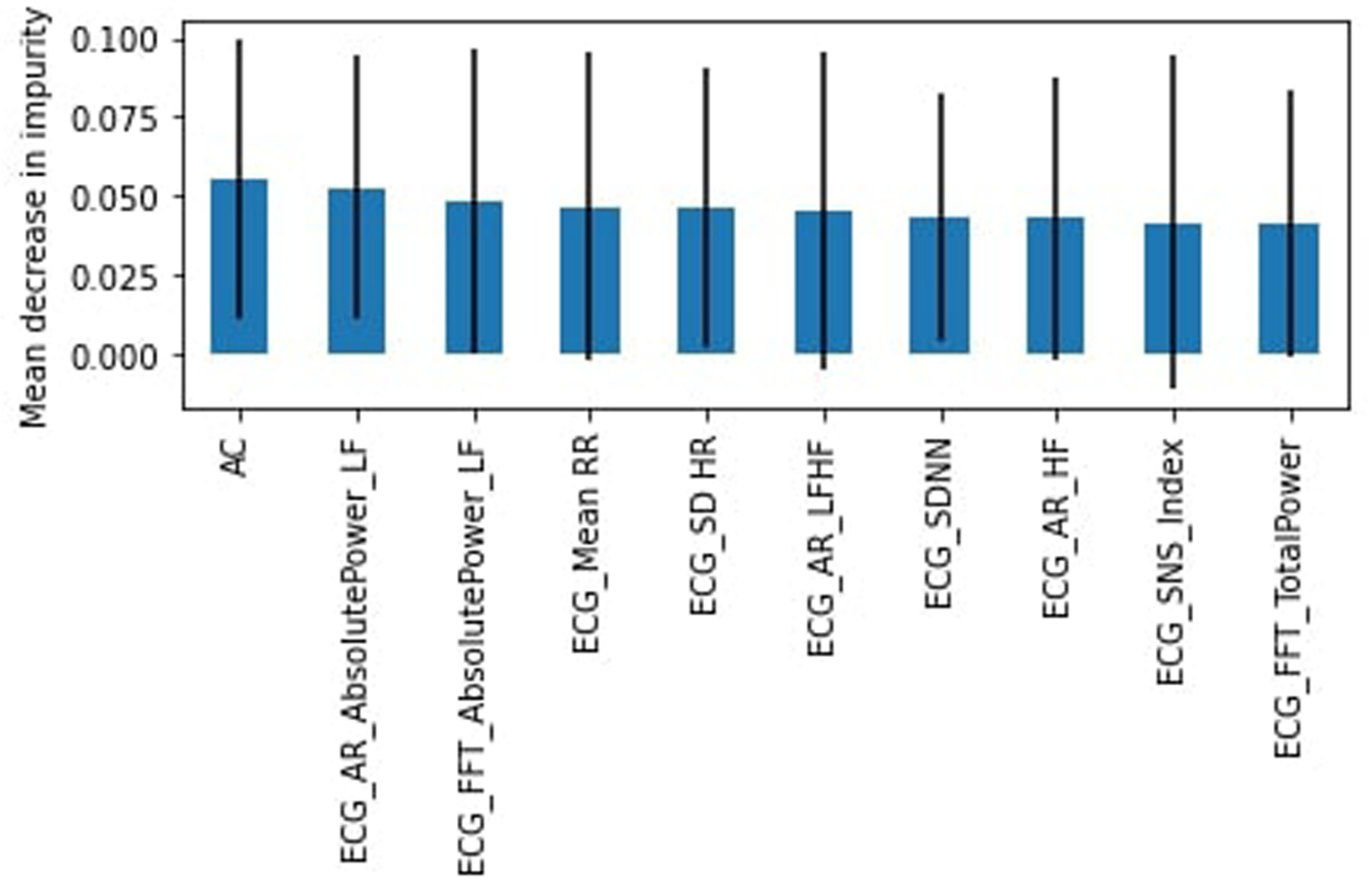
RF feature importance, subjects aged 45–64.

Finally, for the 45–64 years group, the “stress” class comprised 54% of the dataset. We found that the RF model performed better than the SVM with an accuracy of 66% and an F1-score weighted of 64%. The “stress” class had a low recall of 33% and a 53% precision. AC was the most important feature as well (Figure 8).

**Figure 9:**
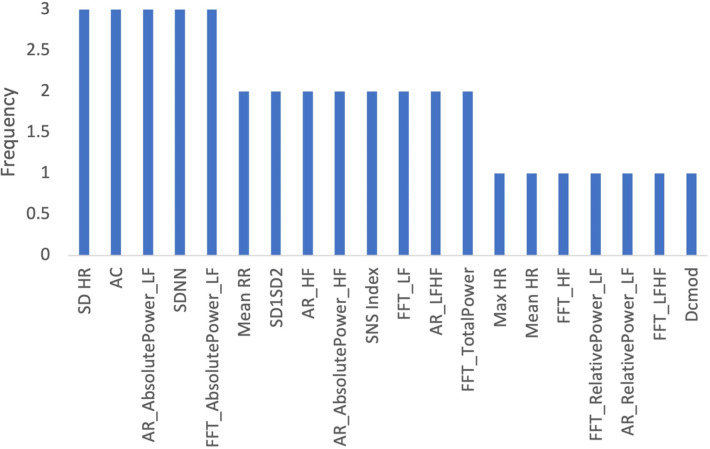
Frequency of features in age related models.

To determine which features were most commonly identified as important across all age groups, we determined the frequency which with features appeared in the top 10. Figure 9 shows that the deviation of the instantaneous heart rate (SD HR), heart acceleration capacity (AC) and AR Low Frequency Absolute Power were the most important features across all age-related models.

### Impact of gender on stress prediction models

Evidence suggests that gender has an impact on HRV (23). To determine whether our stress prediction would improve if we accounted for gender, we trained the RF and SVM learning models for males and females. The “stress” class represented slightly more than 45% of the datasets.

**Figure 10:**
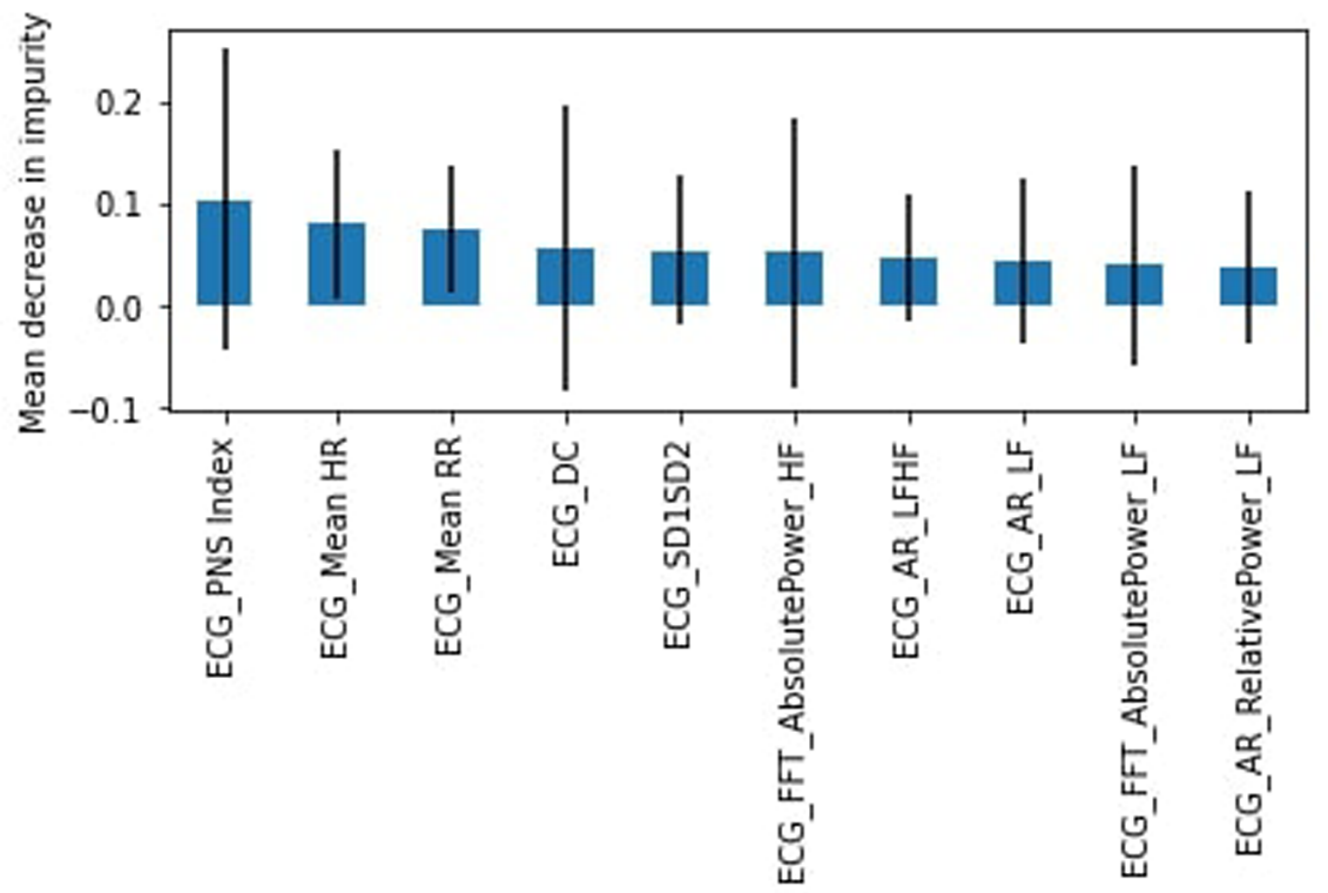
RF feature importance, male participants.

The RF model performed better for the male participants with an accuracy and F1-score weighted of 63%. The precision was 62% and recall was 58% for the “stress” class. The PNS Index was the most important feature (Figure 10).

**Figure 11:**
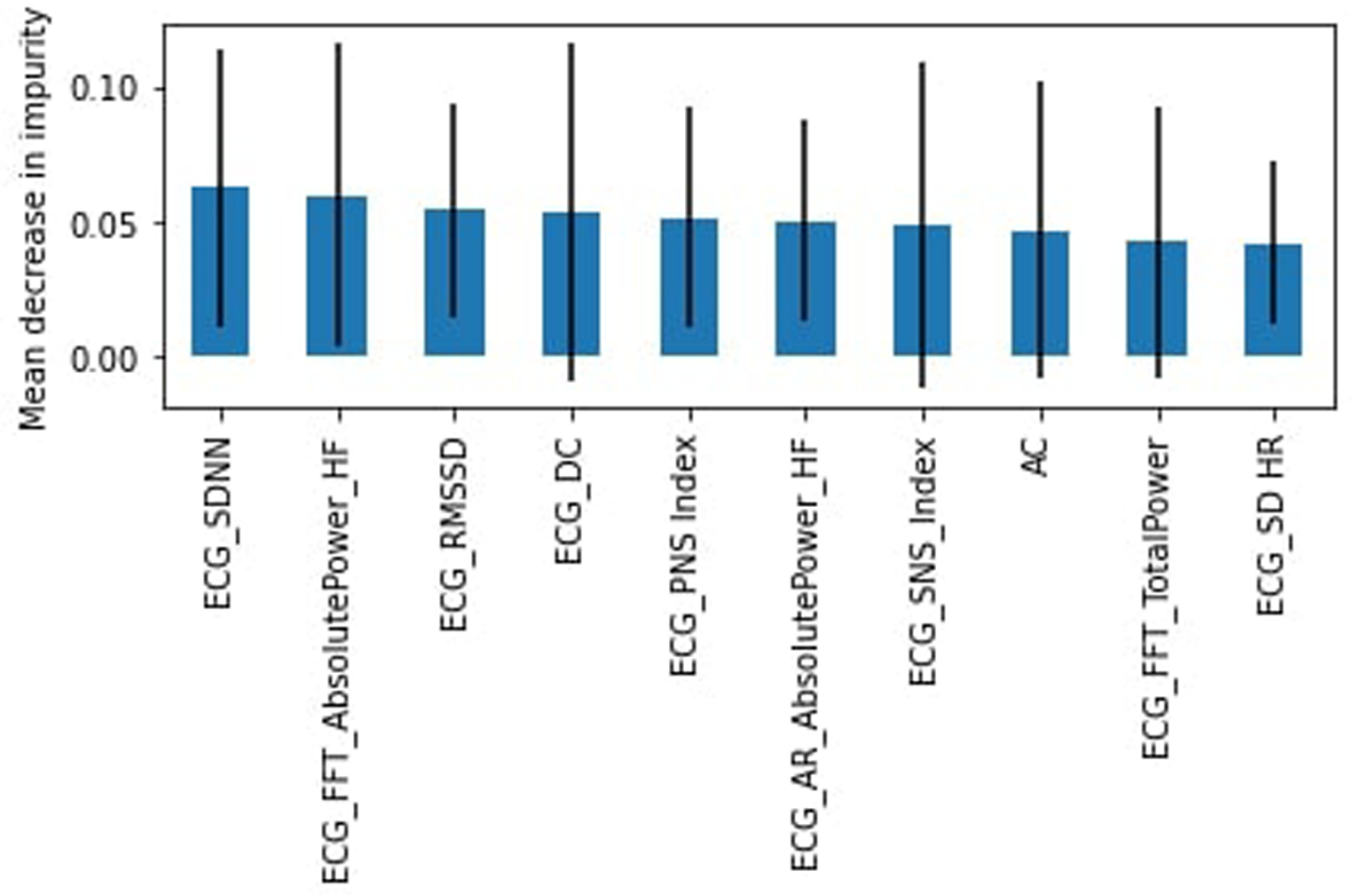
RF feature importance, female participants.

In contrast, the SVM model performed better for female participants with an accuracy of 58% and weighted average of 57%. The “stress” class had a precision of 55% and a recall of 47%. SDNN was found to be the most important feature (Figure 11).

**Figure 12:**
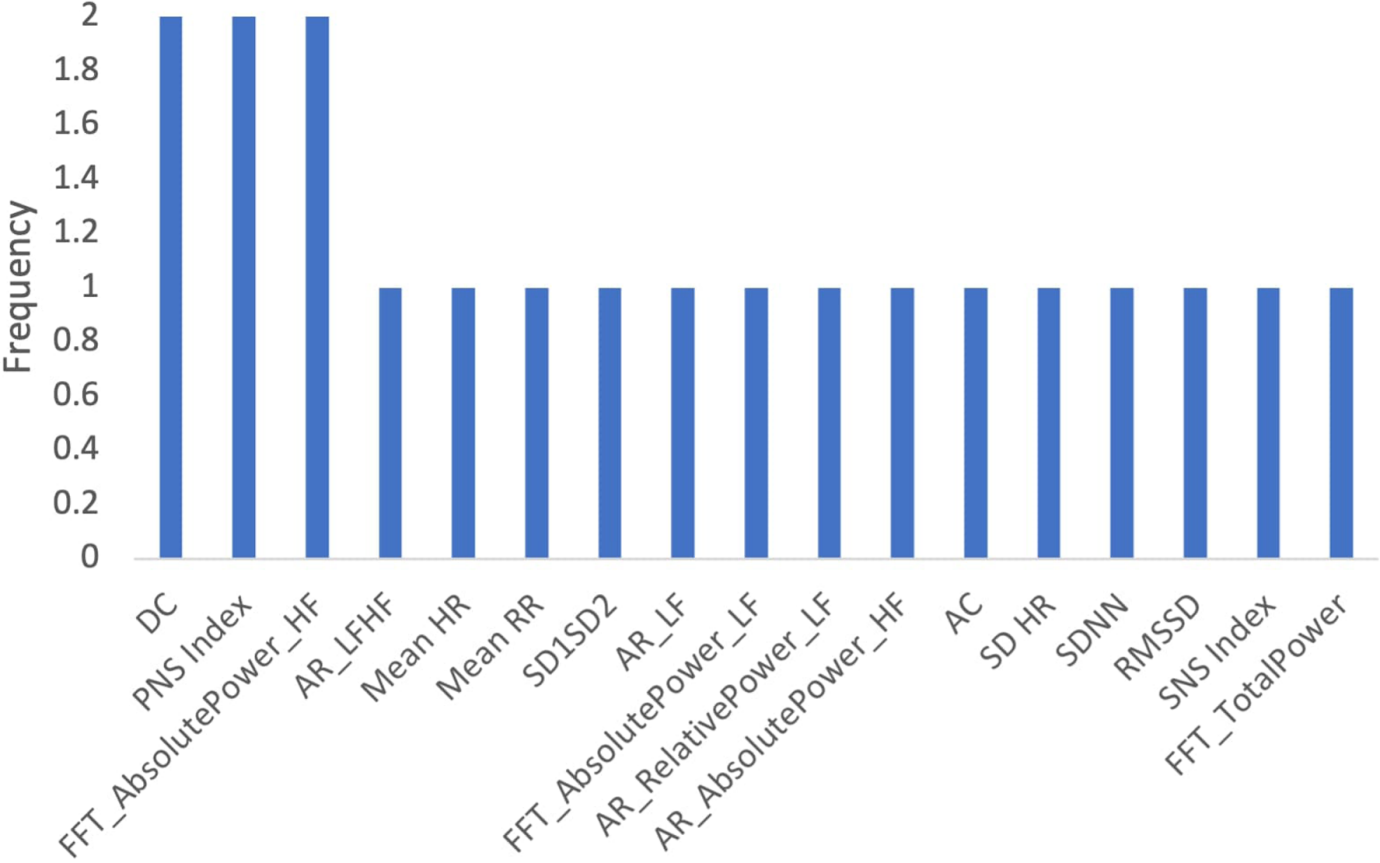
Frequency of Features in gender-related models.

Figure 12 shows the frequency with which each feature appeared as the 10 most important features across both gender-related models. DC, PNS Index, and FFT High Frequency Absolute Power were identified as the most frequently important HRV features.

### Impact of socioeconomic status on stress prediction model

As there are large socioeconomic disparities in cardiovascular disease and HRV, we sought to train our machine learning models based on socioeconomic status (SES) (24). Participants were considered to be in the low SES category if their net income was <30,000 CAD based on an approximation from the Canadian tax cut-off for low-income populations (27). Those with incomes above this threshold were considered medium-to-high SES.

**Figure 13:**
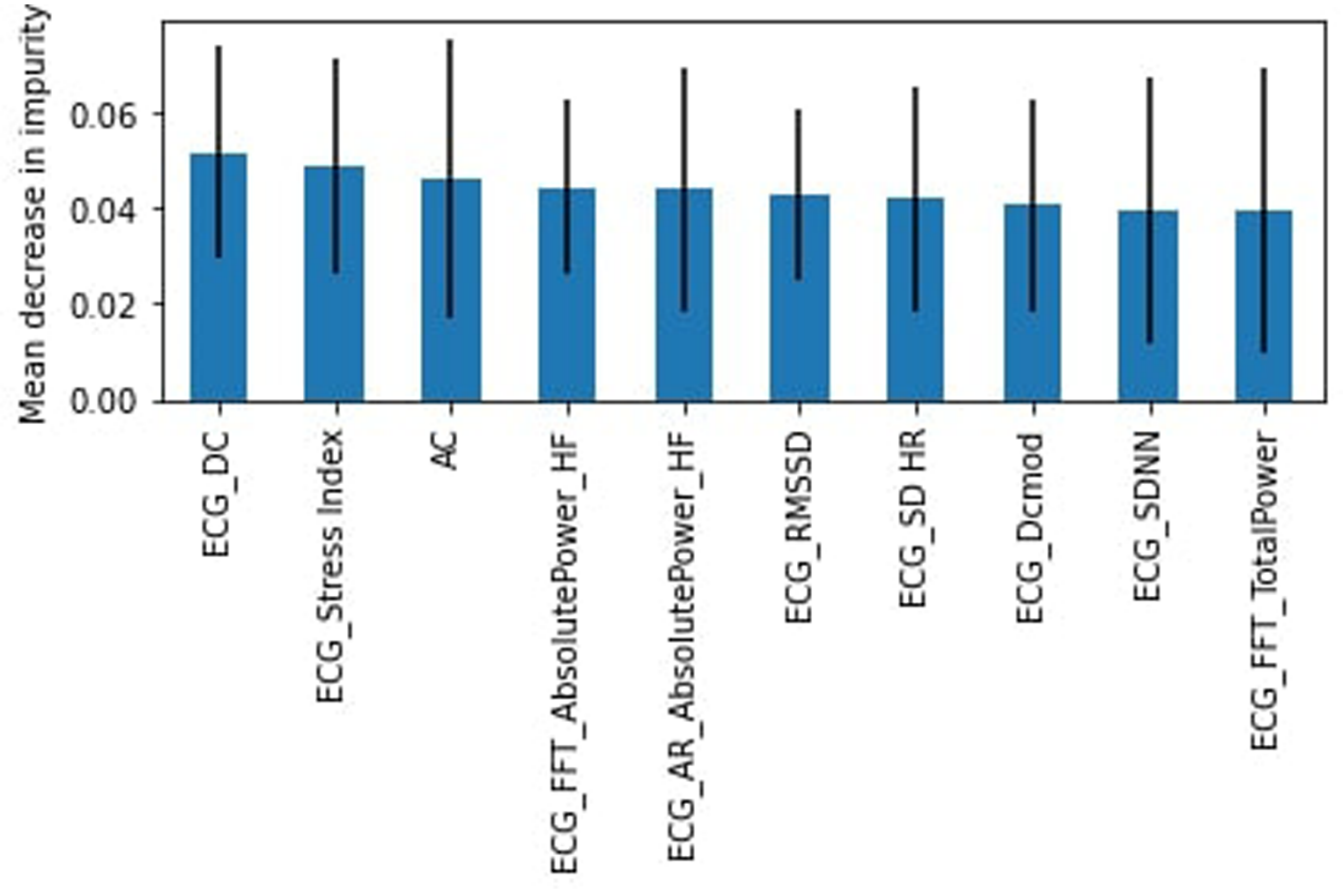
RF feature importance, Low SES participants.

For the low SES group, the “stress” class comprises 40% of the dataset. The SVM model performed better with an accuracy and F1-score weighted of 56%, recall of 45%, and precision of 45% for the “stress” class. The most important feature was DC, the heart deceleration capacity (Figure 13).

**Figure 14:**
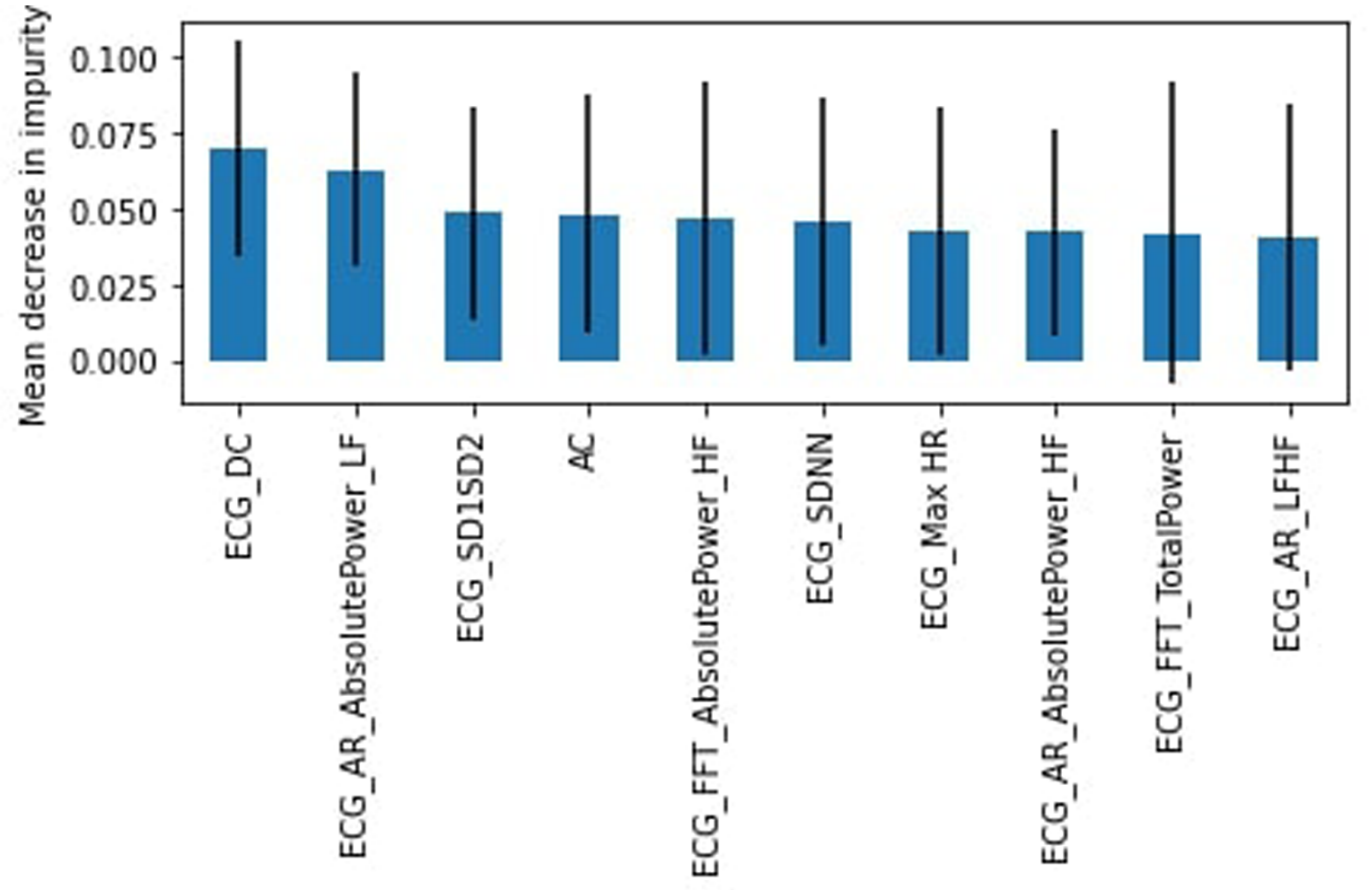
RF feature importance, medium and high SES participants.

For the medium and high SES participants, the “stress” class represented 51% of the dataset. The SVM model performed slightly better than the RF model with accuracy and F1-score weighted of 55%. For the “stress” class, the recall was 62% (slightly higher for RF at 64%) and precision was 55%. DC, the heart deceleration capacity, was again identified as the most important feature (Figure 14).

**Figure 15:**
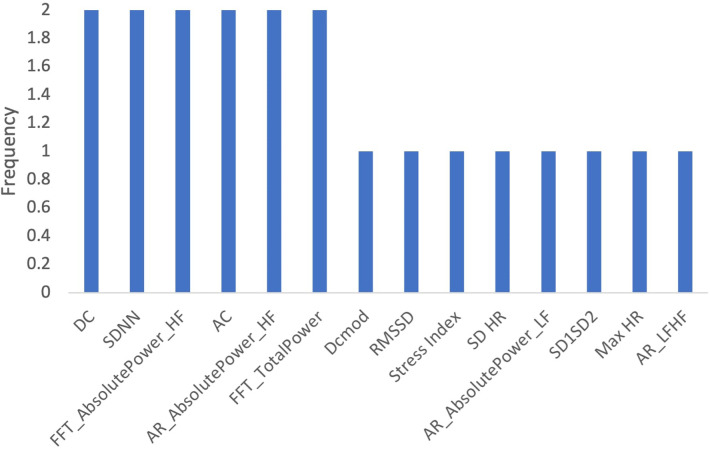
Frequency of features in income-related models.

Figure 15 shows the frequency of features that appeared as the 10 most important features across both income-related models. The most frequently identified features were DC, SDNN, FFT Absolute Power HF, and AC.

### Impact of profession on stress prediction model

Occupational stress is associated with cardiovascular disease and HRV (23). As such, we trained our stress prediction models based on participant occupation. Participants were categorized as workers (full-time, part-time, self-employed, or other) and students. We did not train a model for the retired participant as only one participant was in that category.

**Figure 16:**
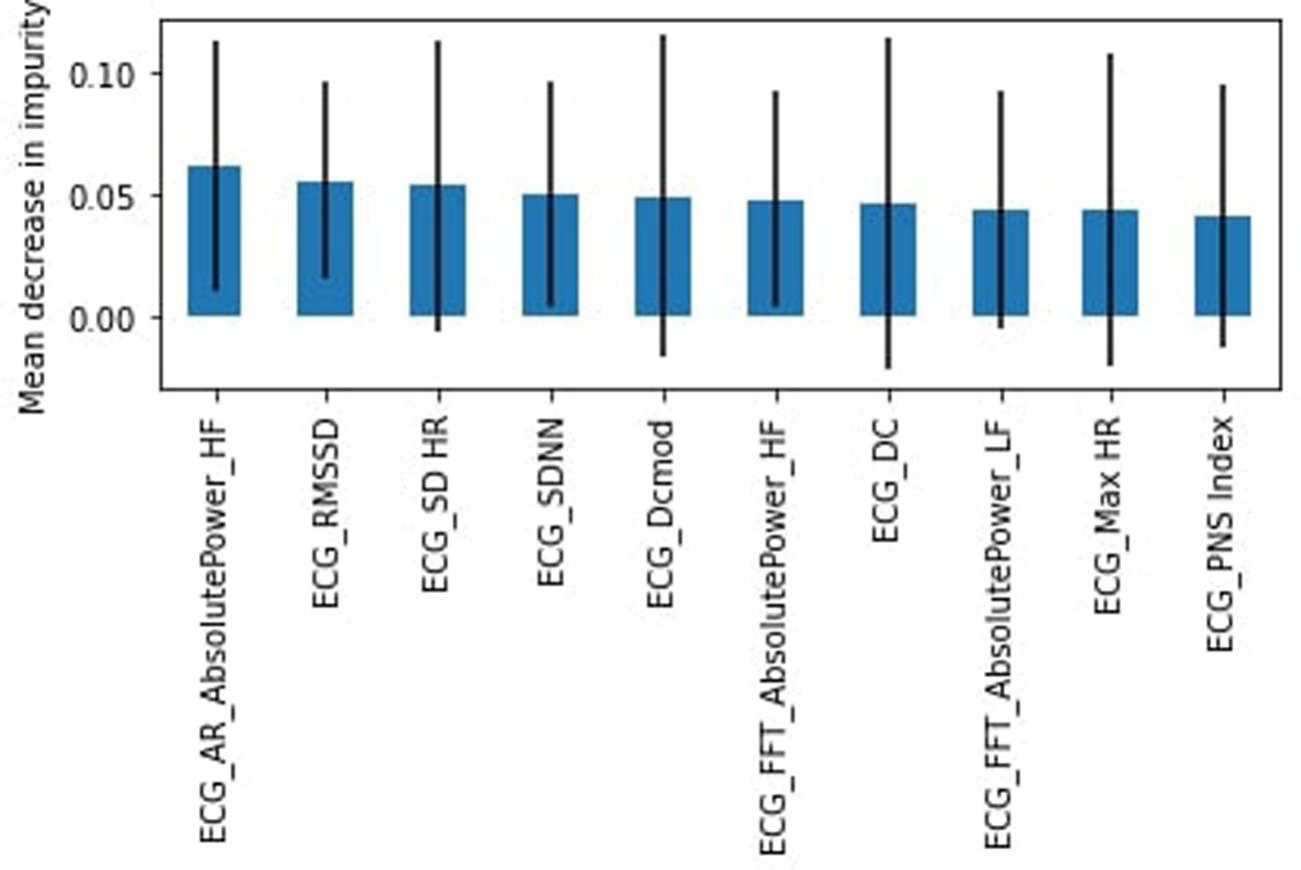
RF feature importance, students.

The SVM had better accuracy (58%) and F1-score weighted (58%) when models were trained for students. The “stress” class represented 49% of the dataset with a recall of 62% and precision of 56%. The AR High Frequency Absolute Power was the most important feature (Figure 16).

**Figure 17:**
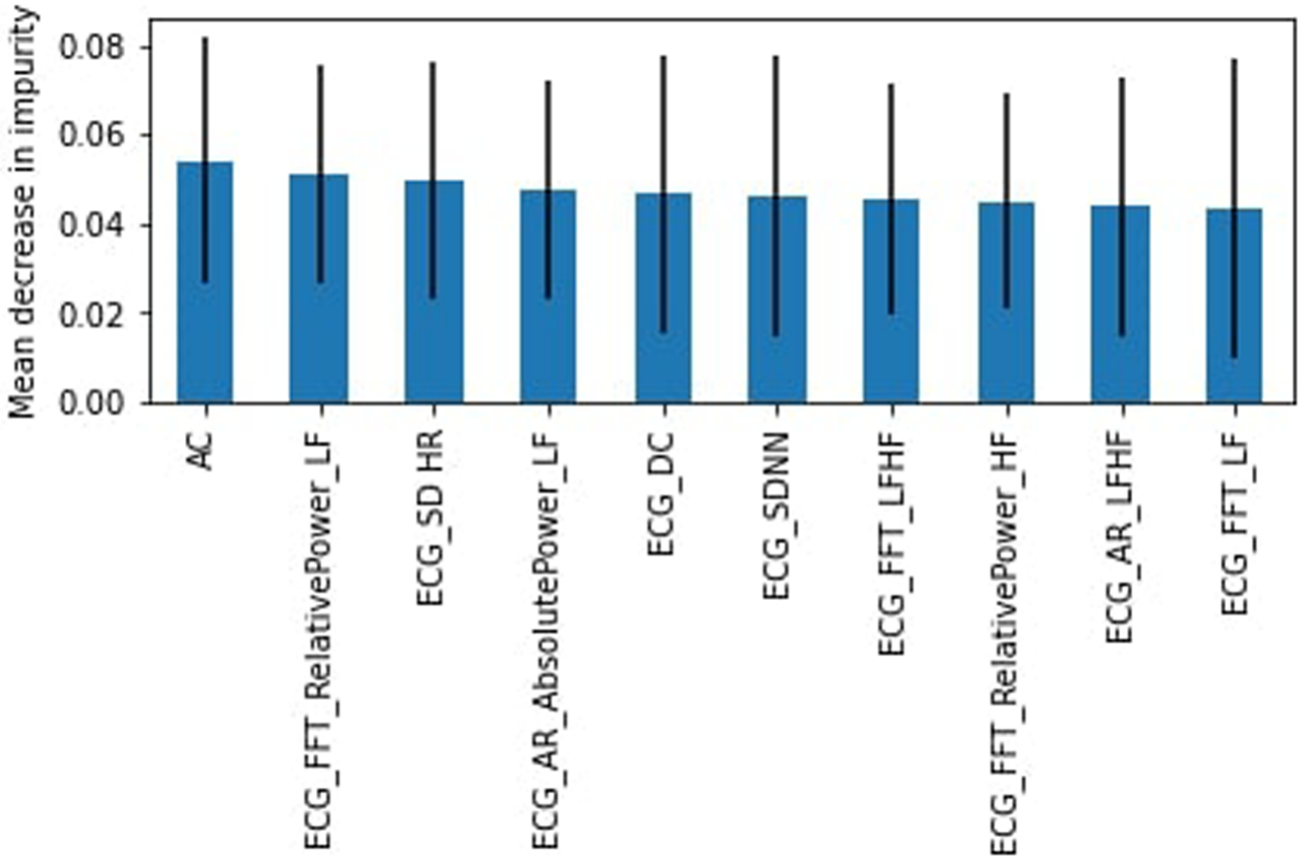
RF feature importance, workers.

When we trained the model for workers, the “stress” class represented 46% of the dataset. The SVM model slightly outperformed the RF in accuracy (55% compared to 53%) and F1-score weighted (54%–52%). The SVM had a better recall (39%) and precision (52%) for the “stress” class. Figure 17 shows the 10 most important features, with the AC as the most important feature.

**Figure 18:**
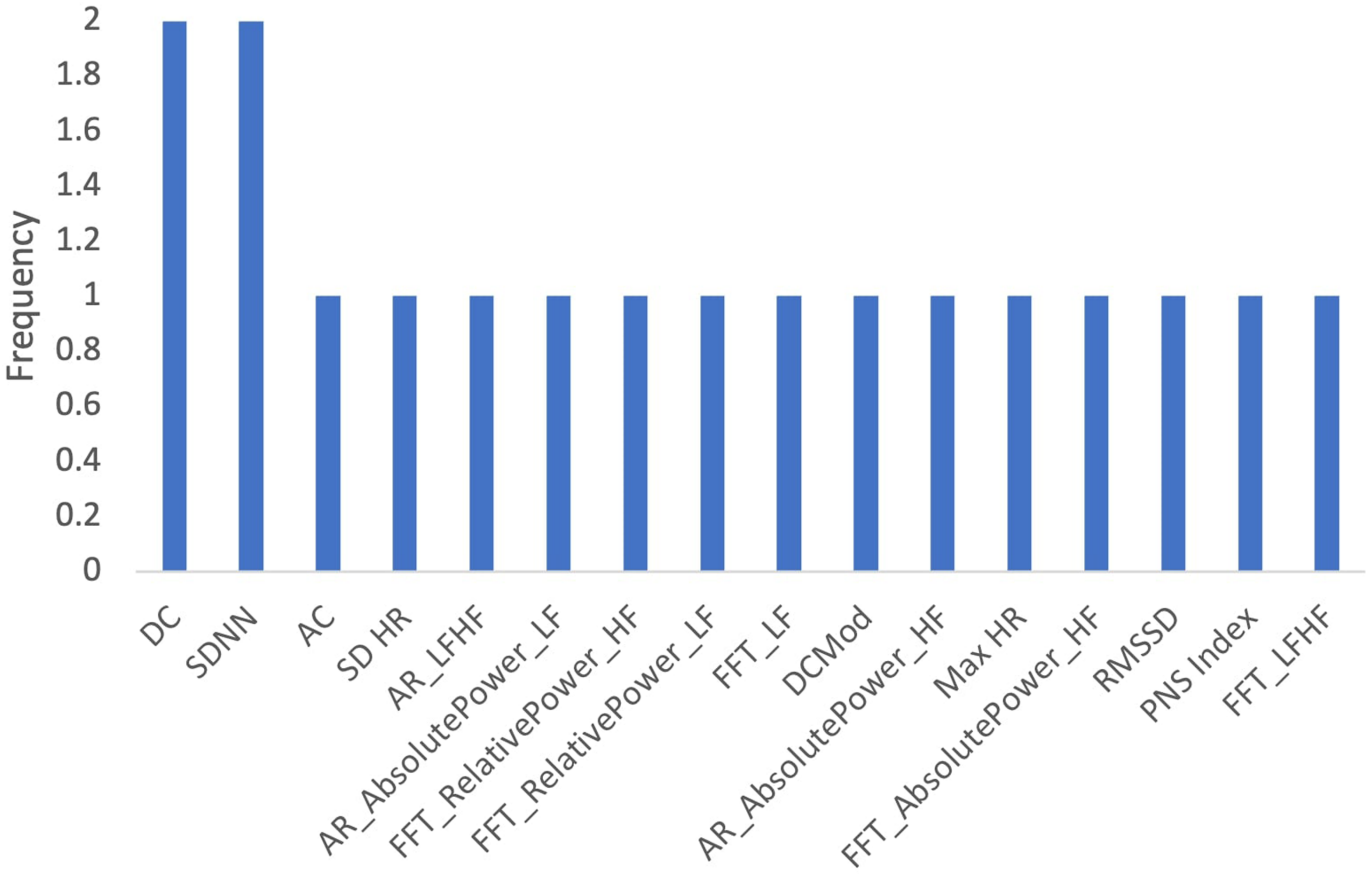
Frequency of features in profession-related models.

We determined which features appeared most frequently as the top 10 most important features across both datasets (Figure 18). The most important features were DC, SDNN, AC, and SD HR.

### Priority HRV features for stress prediction models

**Figure 19:**
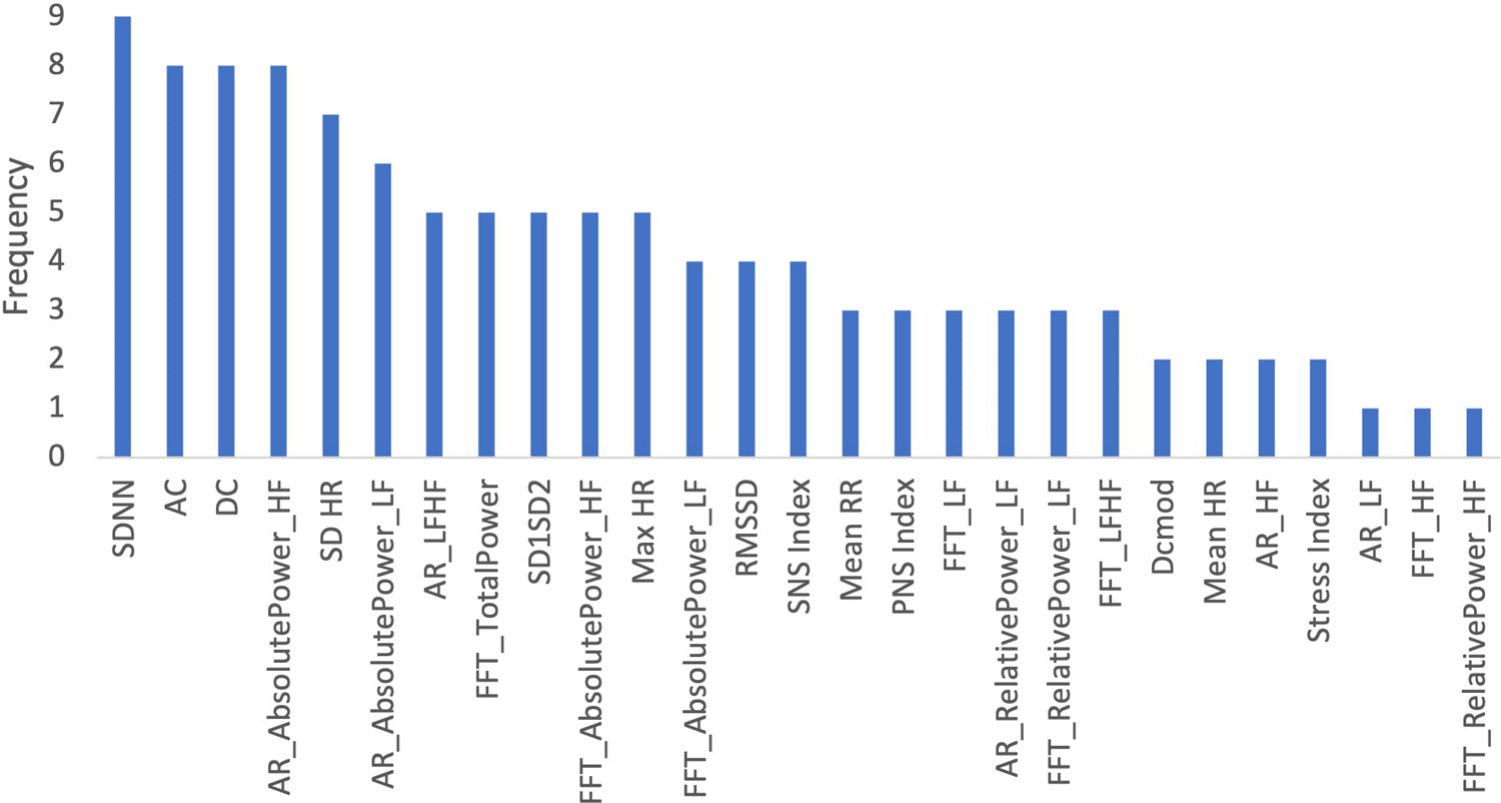
Frequency of features in all models.

Several HRV features collected during the ECG measurements were identified as important across the models trained for the entire dataset, age, gender, socioeconomic status, and profession. We determined the frequency with which each feature appeared as the “10 most important features” across all 10 models described above (Figure 19). The top features identified were SDNN, AC, and DC.

## Discussion

Here we piloted the use of an Apple Watch ECG sensor to predict participant stress levels. Overall, both models performed similarly in different circumstances, achieving F1-weighted scores ranging from 52% to 64%. The state-of-the-art accuracy for stress detection in real-life settings lies approximately between 60% and 80% (12). In general, the “stress” models had a high level of precision but lower recall. The “no stress” models performed generally well with a recall typically above 60%. Considering the ultra-short duration of the ECG measurements performed here compared to the standard, as well as the nature of real-life measurements, the results presented were quite promising.

Divisions by gender, profession or income were found to be good proxies for the prediction models, although more data seems to be needed for improvement. In the majority of cases, the models performed better for the “no stress” class compared to the “stress” class. As the fraction of data falling into the “no stress” class was often greater, the performance discrepancy may be related to class imbalance. Future studies should explore over- and under-sampling techniques to improve the models. Overall, while the models have high specificity, predicting “no stress” states relatively well, they currently lack the predictive power to accurately predict the “stress” states. Future work should focus on frequency-domain metrics and implement novel approaches for data analyses. Additional stress-related variables could also be integrated into the analyses, as well as exploring training and testing datasets based on subjects rather than randomly.

The heart acceleration (AC) and deceleration capacity (DC) were some of the most valuable HRV features included in the model, being present in most, if not all, of the 10 most important features in all models described. This is interesting as AC and DC are relatively new indicators in HRV analyses and lack research with a focus on stress; these results, then, can indicate new avenues of research focusing on these metrics for stress prediction (28). The SDNN, one of the most widely used metrics for time-domain HRV, was also present in most models. Frequency-domain features were commonly identified as important as well. This was consistent with the Task Force recommendations; frequency-domain metrics are better at capturing variations in HRV than time-domain metrics for short measurement periods of time.

Still regarding feature importance, it is important to note the wide error bands for most of the calculated mean decrease in impurity, which points to the fact that the different trees in the random forest models are varied to take into account all complexities in the data. Most features possess similar wide error bands, and that the features described above are repeated throughout the models, suggesting that they are the most important ones and should be evaluated carefully.

One limitation of this study was skewed population representation: participants were primarily white females. As such, there may have been insufficient data to accurately train the models for other representative groups. As well, due to limited participant numbers, it was challenging to stratify characteristics. For example, socioeconomic status and profession were only stratified into two categories which may be insufficient to capture demographic features. Here we applied the use of RF and SVM to train the models (12), however, other methods may perform better including Deep Learning approaches. Future work could apply Deep Learning methods using the raw signals from each participant's ECG measurements as time series data.

To the best of our knowledge, this is the first study to use Apple Watch ECG data to predict the stress levels of individuals. The results are currently in the low-end of state-of-the-art; as mentioned above, stratifying participants can improve accuracy, and larger studies that allow further stratification of the cohort might achieve even better results. In addition, data was collected in real-life conditions which can potentially introduce noise in the data. On the same token, stress self-report was used as the ground truth for a given moment in time, which might not always reflect physiological parameters. Since the results were promising with these factors potentially introducing noise in the data, and given the novelty of the data type, conducting further studies in a controlled setting, such as applying stressors in a lab environment, could give us additional insights into the relationship between Apple Watch ECG data and stress. In addition, since the Apple Watch can also collect additional data such as sleep and physical activity, it should also be interesting to use ECG data with other stress-related variables, as they can complement the data and increase the models' predictive power.

## Conclusion

This study presented an analysis of Apple Watch ECG data from 33 participants. To the best of our knowledge, this is the first study to use Apple Watch ECG data to predict stress levels of individuals. RF and SVM models were developed for the task, with the models performing similarly.

Further, the results are in line with the start-of-the-art for stress prediction, although at the low-end. This is very promising considering the ultra-short-term and real-life nature, as well as the novelty of, the Apple Watch ECG data. However, while the current models have high specificity, predicting “no stress” states relatively well, it lacks the predictive power to accurately predict the “stress” states as of yet. Future work should focus on the AC, DC, SDNN as well as frequency-domain metrics and implement novel approaches for data analyses, such as Deep Learning, as well as integrating additional stress-related variables into the analyses.

Overall, the results from the pilot study validate the continued development of wearable ECG technology and suggest that, with further refinement, models can likely achieve stress prediction with state-of-the-art quality. In that way, we can develop near real-time, non-intrusive stress detection, monitoring, and intervention applications using a technology that is already widely popular and accepted by the population, leading to better health outcomes.

## Data Availability

'The original contributions presented in the study are included in the article/**Supplementary Materials**, further inquiries can be directed to the corresponding author/s.
