# Supplementary material for: Using apple watch ECG data for heart rate variability monitoring and stress prediction: A pilot study
Source: Front Digit Health. 2022 Dec 9;4:1058826. doi: 10.3389/fdgth.2022.1058826 (PMC9780663; doi:10.3389/fdgth.2022.1058826)
Supplement: Supplementary file 1 [file Datasheet1.docx]

# ECG Instructions

Preparing for taking the reading:

- Rest your arms on a table or in your lap while you take a recording. Try to relax and not move too much.
- Make sure that your Apple Watch isn’t loose on your wrist. The band should be snug, and the back of your Apple Watch needs to be touching your wrist.
- Make sure that your wrist and your Apple Watch are clean and dry.
- Make sure that your Apple Watch is on the wrist that you selected in the Apple Watch app. To check, open the Apple Watch app, tap the My Watch tab, then go to General > Watch Orientation.
- Move away from any electronics that are plugged into an outlet to avoid electrical interference.

Please follow the instructions below when taking an ECG reading:

1. Make sure that your Apple Watch is snug and on the wrist that you selected in the Apple Watch app. To check, open the Apple Watch app, tap the My Watch tab, then go to General > Watch Orientation.
2. Open the ECG app 
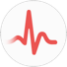
 on your Apple Watch.
3. Rest your arms on a table or in your lap.
4. With the hand opposite your watch, hold your finger on the Digital Crown. You don't need to press the Digital Crown during the session.
5. Wait. The recording takes 30 seconds.


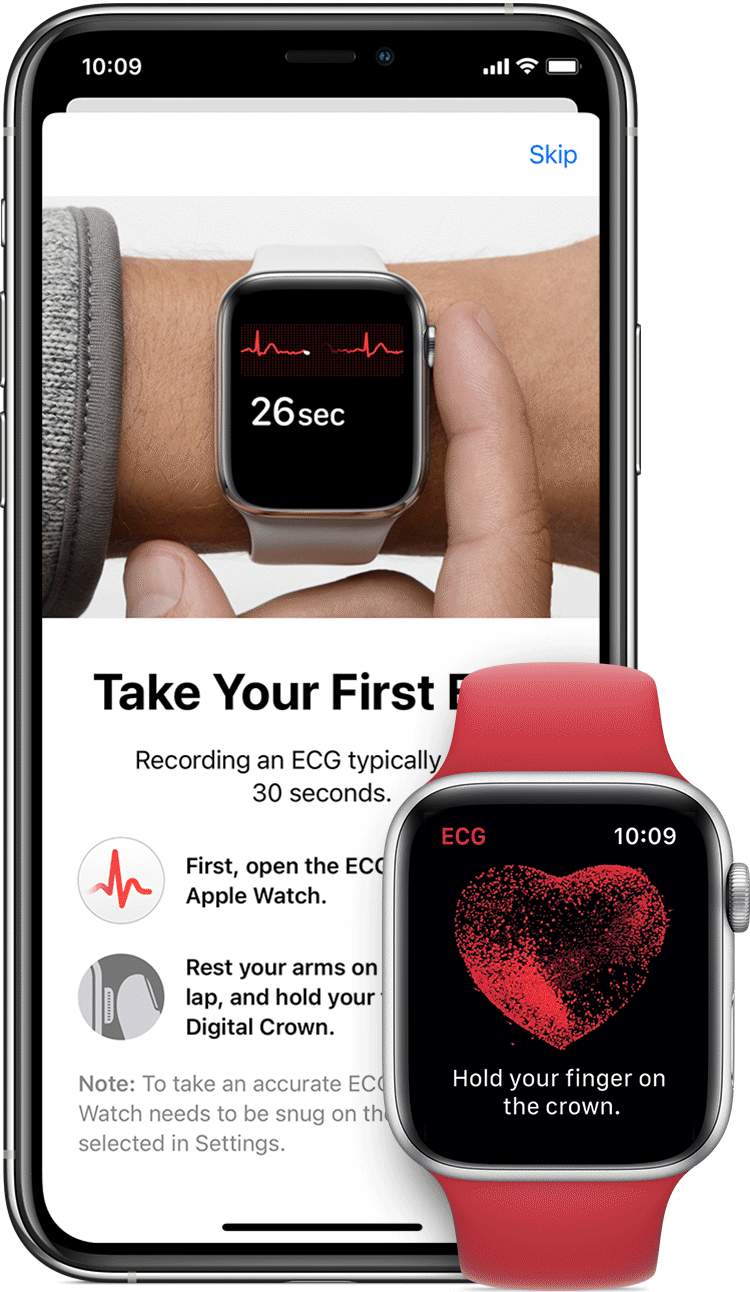


**Please keep in mind that the Apple Watch cannot: detect a heart attack; blood clots or stroke; other heart-related conditions. If you are not feeling well, please contact emergency services.**

Adapted from: <https://support.apple.com/en-ca/HT208955>
